# Bartter syndrome-like phenotype in a patient with diabetes: a case report

**DOI:** 10.1186/s13256-018-1752-6

**Published:** 2018-08-17

**Authors:** Chamara Dalugama, Manoji Pathirage, S. A. M. Kularatne

**Affiliations:** Department of Medicine, University of Peradeniya, Peradeniya, Sri Lanka

**Keywords:** Hypokalemia, Metabolic alkalosis, Bartter’s syndrome, Acquired, Idiopathic

## Abstract

**Background:**

Bartter’s syndrome is a rare genetic tubulopathy affecting the loop of Henle leading to salt wasting. It is commonly seen *in utero* or in early neonatal period. Rare cases of acquired Bartter’s syndrome are reported in association with infections like tuberculosis, granulomatous conditions like sarcoidosis, autoimmune diseases, and drugs. The mainstay of management includes potassium, calcium, and magnesium supplementation.

**Case presentation:**

We report the case of a 62-year-old Sri Lankan Sinhalese man with diabetes and hypertension presenting with generalized weakness with clinical evidence of proximal myopathy. He was severely hypokalemic with high urinary potassium excretion and hypochloremic metabolic alkalosis. He poorly responded to intravenously administered potassium supplements. A diagnosis of idiopathic Bartter-like phenotype was made. He responded well to spironolactone and indomethacin.

**Conclusions:**

Patients presenting with body weakness need serum potassium estimation. Acquired Bartter’s syndrome although rare, should be ruled out in those with hypokalemia and metabolic alkalosis with increased urinary potassium loss with poor response to potassium replacement.

## Background

Bartter’s syndrome (BS) is a rare genetic condition described by Bartter and coworkers in 1962. It is a tubulopathy affecting the thick ascending limb of loop of Henle (LOH) leading to salt wasting. It is characterized by hypokalemia, hypocalcemia, hypercalciuria, metabolic alkalosis, hyperreninemic hyperaldosteronism, and a normal blood pressure [1–4]. It is commonly seen in the prenatal and neonatal period. Very few cases of acquired BS are described in the literature; they are commonly associated with tuberculosis, autoimmune conditions, sarcoidosis, or following administration of drugs such as aminoglycosides [5–12]. We report a case of idiopathic BS-like phenotype in a man with diabetes.

**Table 1:** Investigation findings of the patient

Serum calcium	1.81 mmol/L	(2.2–2.6)
Serum chloride	93 mmol/L	(102–109)
Serum magnesium	0.73 mmol/L	(0.73–1.06)
Serum osmolality	282 mOsmol/kg	(275–295)
Urine potassium	30 mmol/L	< 25
Urine PH	7.2	
Urine calcium creatinine ratio	0.47	
Thyroid-stimulating hormone	1.5 u/L	(0.4–5.0)

**Table 2:** Acquired causes of Bartter’s syndrome

Drugs	
1. Aminoglycosides (gentamicin, capreomycin and viomycin)	
2. Loop diuretics	
3. Colistin	
4. Amphotericin B	
Chronic infections	
Tuberculosis	
Autoimmune conditions	
Sjögren’s disease	
Granulomatous conditions	
Sarcoidosis	

## Case presentation

A 62-year-old Sri Lankan Sinhalese man from the North Central Province of Sri Lanka presented with generalized malaise and body weakness. He had type 2 diabetes and had been on Mixtard (human insulin) for 10 years. He had been hypertensive for 5 years and was on losartan potassium. His anti-hypertensive drugs were withheld 2 months previously because he had low-normal blood pressure. He described proximal muscle weakness of the body of 1 month’s duration with difficulty in getting up from a squatting position and raising his hands above his head. He noticed polyuria and nocturia with recent worsening of glycemic control. There was no history of fever, vomiting, diarrhea, or any drug abuse prior to the onset of the symptoms. He denied a suggestive family history of diabetes mellitus, hypertension, or renal disease.

On examination, he was conscious and rational. His blood pressure was 110/64 mm Hg and his pulse was 76/minute. The rest of the cardiovascular system and respiratory system examination was normal. His abdomen was soft and non-tender. A neurological examination revealed normal higher functions and cranial nerves. A motor system examination showed hypotonia of all four limbs and a power of 4/5 in both lower limbs and 5/5 in both upper limbs. All reflexes were present, but diminished. His plantar reflex was bilaterally unresponsive. There was no sensory or autonomic involvement.

Initial blood investigations showed sodium ion (Na+) 146 mEq/L, potassium ion (K+) 1.95 mEq/L, urea 4.3 mmol/L, creatinine 0.7 mg/dl, and random blood glucose 300 mg/dl. His hemoglobin was 13.2 g/dL with white count of 5.7 × 10^6^ and platelets 240 × 10^6^. Transaminases were normal. His serum albumin was 34 g/L. Severe hypokalemia was confirmed in the repeat blood sample. Arterial blood gas revealed severe metabolic alkalosis with pH of 7.6, partial pressure of carbon dioxide (CO_2_) of 41 mmHg, and bicarbonate of 40.3 mmol/L.

Further investigations revealed the following (Table 1). The results of an X-ray of his kidney-ureter-bladder and an ultrasound scan of his kidneys were normal with no evidence of nephrocalcinosis.

He was treated aggressively with intravenously administered potassium chloride, and calcium and magnesium supplements. But he was noted to have persistent hypokalemia with pottasium wasting in urine. Spironolactone was added to the treatment regime. On day 4 while receiving potassium chloride and spironolactone, his serum potassium was 2.6 mmol/L. In this clinical context BS was suspected in our patient. Unfortunately, he could not afford plasma rennin and serum aldosterone levels. He was started on indomethacin 50 mg thrice a day. On day 7 he was noted to have a marked improvement in proximal muscle weakness and his serum potassium reached 3 mmol/L; with the correction of potassium, our patient’s glycemic controlled improved. He was discharged with the advice of liberal salt intake, K+ and magnesium cation (Mg2+) supplements, spironolactone, and indomethacin. He is currently doing well with low normal potassium value with the above treatment.

## Discussion

BS is a rare autosomal recessive condition first described by Bartter and his coworkers in 1962 [1]. BS results from mutations affecting any of the five ion transport proteins in the thick ascending LOH, giving a clinical picture of salt wasting and hypokalemic metabolic alkalosis. In a report from the Framingham Heart Study, the prevalence of BS was 1 in 1,000,000 [2].

Biochemical abnormalities include hypokalemia, hyponatremia, mild hypomagnesemia, hypocalcemia, hypochloremic metabolic alkalosis, and reduced urine concentrating ability with increased urinary excretion of calcium and prostaglandins. Patients tend to have normal or low blood pressure. Nephrocalcinosis is a known association with long-standing BS. Plasma rennin and aldosterone levels are increased [3].

BS closely mimics the effects of chronic ingestion of a loop diuretic. Defects in the transport proteins of the thick ascending limb of LOH lead to loss of luminal-positive electrical transport potential which drives the paracellular reabsorption of sodium, calcium, and magnesium. It leads to loss of sodium, chloride, calcium, and magnesium in urine. Loss of sodium and chloride along with water with volume contraction lead to activation of rennin-angiotensin-aldosterone (RAA) pathway [4].

BS can occur due to various genetic defects leading to reduced activity of one of the several electron transporters in the thick ascending limb of LOH. Five main genetic defects are described. Defective function of the Na-K-2Cl cotransporter in the luminal membrane [5], the luminal potassium channel [6], and the basolateral chloride channel [7] are the causes of BS types I, II, and III, respectively. Type IV BS is associated with sensorineural deafness due to reduced activity of both ClC-Ka and ClC-Kb transporters [8]. Defects in the calcium-sensing receptor (CaSR) in the basolateral membrane of the thick ascending limb can impair sodium chloride transport and generate a mild Bartter phenotype called type V [9]. BS is commonly seen in the prenatal or neonatal period. Acquired BS is very rare with few case reports in the medical literature (Table 2). Chronic diuretic abuse with a loop diuretic is well known to cause BS-type phenotype [3]. Pulmonary tuberculosis [10] and antituberculous medications (capreomycin and viomycin) are known to cause a BS-like phenotype. Several drugs are reported to produce a BS-like phenotype, including aminoglycosides [11, 12], colistin [13], and amphotericin B [14]. Acquired BS is reported in autoimmune conditions such as Sjögren’s disease [15] and granulomatous conditions like sarcoidosis [16, 17].

Whether BS and diabetes mellitus are causally associated is unknown. In the literature a few case reports describe idiopathic BS-like phenotype in association with type 2 diabetes. Venkat *et al.* described patients with BS who displayed elevated insulin concentrations, with hyperplasia of the islets of Langerhans noted at autopsy [18]. Worsening of glycemic control following a diagnosis of late onset BS was described in another case report [19]. However, insulin secretion is decreased in hypokalemia [20]. Treatment with potassium supplements in BS improves glycemic control.

Our patient presented with worsening glycemic control with generalized weakness of the body with malaise. He was found to be severely hypokalemic with pottasium wasting in urine and metabolic alkalosis. We could not find any association with other conditions, including tuberculosis, sarcoidosis, or autoimmune conditions. He was not on long-term loop diuretics or other culprit drugs such as aminoglycoside and cisplatin. He had no past exposure to heavy metals. So a diagnosis of idiopathic acquired BS-like phenotype was made.

The mainstay of treatment in BS is correction of hypokalemia and alkalosis. Doses of potassium chloride supplementation should individually be titrated in accordance to the patient’s needs and balanced by the renal loss [21]. Potassium-sparing agents such as spironolactone or triamterene would be an effective additive to supplementation since administered potassium is lost through the kidney in a short period of time. The most widely used group of medications in the treatment of classic BS is the prostaglandin synthetase inhibitors. Indomethacin, aspirin, and ibuprofen have all been tried and the best evidence comes from indomethacin [22]. As potassium wasting can be exaggerated by magnesium deficiency, there is a need to correct magnesium deficiency. In adults, the use of angiotensin-converting enzyme inhibitors (captopril, enalapril) has had conflicting results [23]. The use of selective and specific cyclooxygenase-2 (COX-2) inhibitors has been described as a successful treatment option in treating BS [24].

Our patient was initially treated with large intravenously administered doses of potassium chloride with orally administered potassium supplementation. Magnesium and calcium salts were supplemented. He was treated with large doses of spironolactone and indomethacin. With the treatment his potassium level increased to 3 mmol/L, his glycemic control improved, and a marked improvement in weakness was noted.

## Conclusions

Hypokalemia should be ruled out when patients present with generalized weakness of the body. Acquired BS should be considered in an adult with hypokalemia and hypochloremic metabolic alkalosis with increased urinary potassium loss.
